# Abundance, Diversity and Metabolomic Profiles of Soil Cyanobacteria‐Dominated Microbial Communities Under Long‐Term Differential Management in an Olive Orchard

**DOI:** 10.1111/1462-2920.70384

**Published:** 2026-07-15

**Authors:** Mohammad Yaghoubi Khanghahi, Rosangela Addesso, Luigi Lucini, Leilei Zhang, Francesco Maria Calabrese, Margherita Chiarini, Matteo Bernardi, Lisa Signorile, Alba N. Mininni, Bartolomeo Dichio, Carmine Crecchio, Maria De Angelis, Mario De Tullio, Adriano Sofo

**Affiliations:** ^1^ Department of Agricultural, Forestry, Food and Environmental Sciences (DAFE) Università Degli Studi Della Basilicata Potenza Italy; ^2^ Department of Soil, Plant and Food Sciences University of Bari “Aldo Moro” Bari Italy; ^3^ Department for Sustainable Food Process Università Cattolica del Sacro Cuore Piacenza Italy; ^4^ Independent Researcher, National Geographic Rome Italy; ^5^ Department of Earth and Geoenvironmental Sciences University of Bari “Aldo Moro” Bari Italy

**Keywords:** agroecosystem biodiversity, autotrophic microorganisms, soil cyanobacteria, soil fertility, sustainable management

## Abstract

This study assessed the effects of 22 years of sustainable (*S*_mng_) versus conventional (*C*_mng_) management on cyanobacteria‐enriched microbial communities in a Mediterranean olive orchard. Surface soils were selectively cultivated in nitrogen‐free Bristol medium to obtain cultivable cyanobacteria‐dominated, nitrogen‐fixing assemblages for molecular and metabolomic analyses. Cyanobacteria‐enriched assemblages derived from *S*_mng_ soils showed greater phylogenetic diversity and distinct community clustering under N‐free cultivation conditions. At the cyanobacterial genus level, *C*_mng_‐derived communities were dominated by *Leptolyngbya*, whereas *S*_mng_ assemblages shifted towards *Nodosilinea*, alongside the consistent presence of heterocystous Nostoc. These patterns suggest that reduced disturbance and organic inputs favour the enrichment of stable cultivable nitrogen‐fixing cyanobacteria. Untargeted metabolomics identified 494 features and revealed strong functional divergence. *C*_mng_‐derived assemblages showed enhanced lipid and vitamin biosynthesis, consistent with stress‐related metabolic investment. In contrast, *S*_mng_‐derived assemblages displayed increased amino acid biosynthesis and enhanced activity of signalling‐ and hormone‐related metabolic pathways, including cytokinin biosynthesis and ethylene biosynthesis‐related pathways. These metabolic signatures suggest a shift towards resource allocation supporting microbial interactions, plant signalling and nitrogen transfer within the soil–plant system. Overall, the results indicate that *S*_mng_ favours cyanobacteria‐dominated communities with metabolic traits linked to ecosystem functioning, underscoring their role in improving soil quality and resilience in semi‐arid agroecosystems.

## Graphical abstract

Sustainable management in a semi‐arid olive orchard increased abundance and diversity of nitrogen‐fixing cyanobacteria, shifting dominance towards *Nodosilinea* and *Nostoc*, while conventional soils were dominated by *Leptolyngbya*. Metabolomics showed stress‐related lipid and vitamin production under conventional management versus enhanced amino acids and hormone‐related metabolites under sustainable practices, supporting soil resilience.

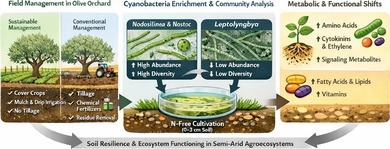

## 1 Introduction

Photoautotrophic prokaryotic cyanobacteria (blue–green algae) are integral components of soil microbial communities. Through oxygenic photosynthesis, they are among the principal primary producers of organic matter and biologically available nitrogen, playing a crucial role in both terrestrial and aquatic food webs (Saleem et al. 2025). Occupying similar ecological niches, cyanobacteria occur in unicellular and multicellular filamentous forms and are widely distributed in surface soils worldwide, including extreme environments such as hot and cold deserts, Arctic and Antarctic regions and highly acidic habitats. Accordingly, cyanobacteria encompass thermophilic, psychrophilic, halophilic, acidophilic, alkaliphilic and radiation‐resistant taxa (Nowruzi and Alibabaei 2026; Cano‐Díaz 2026). While cyanobacterial densities as high as 10^8^ cells per gram of soil have been documented, typical soils generally sustain populations ranging from 10^3^ to 10^4^ cells per gram (Metting 1981). Cyanobacteria are primarily concentrated in the upper soil layers (from a few millimetres in fine‐textured soils to several centimetres in coarse soils), where light availability supports photosynthetic activity. Nevertheless, some species appear capable of limited heterotrophic metabolism in deeper aphotic zones, possibly following downward transport by rainfall (Addesso et al. 2024).

In the current global context, increasing food demand driven by population growth and the urgent need for sustainable agricultural practices to replace conventional systems associated with significant environmental impacts, most notably soil degradation, pose major challenges (Jamali et al. 2021). In this framework, cyanobacteria offer substantial potential benefits for the development of resilient and sustainable agroecosystems, particularly under conditions further exacerbated by climate change (Nawaz et al. 2025; Saleem et al. 2025). Their role is particularly significant in semi‐arid and arid regions, where soils are often poor in organic carbon and exposed to high temperatures and intense solar radiation. Carbon dioxide fixed through cyanobacterial photosynthesis is returned to the soil following cell senescence, contributing to biomass accumulation and organic carbon enrichment. This process enhances soil aggregation, porosity, water‐holding capacity and nutrient solubilisation, thereby improving soil fertility and resistance to erosion (Lucius and Hagemann 2024; Pandey et al. 2025). Moreover, nitrogen‐fixing cyanobacteria provide a natural and cost‐effective source of nitrogen, a major limiting nutrient in agricultural systems (Gonçalves 2021; Tan et al. 2021).

Moreover, cyanobacteria are regarded as pioneer organisms in terrestrial ecosystems and are fundamental to the establishment of the present oxygen‐rich atmosphere; they also play a key role in the formation and stabilisation of biological soil crusts (Samolov et al. 2020; Chittora et al. 2020; Novakovskaya et al. 2022) and engage in synergistic biotic interactions with other soil microorganisms (Solomon et al. 2023; Zhou et al. 2024), facilitating nutrient exchange and contributing to the creation of a microenvironment conducive to plant development. Cyanobacteria can produce a wide range of bioactive compounds, including extracellular polysaccharides, phytohormones, antibiotics and antifungal metabolites, which promote plant growth and contribute to the suppression and biocontrol of plant pathogens (Teplitski and Rajamani 2010).

Due to their metabolic, biochemical and morphological plasticity in response to environmental fluctuations, cyanobacteria can rapidly activate specific regulatory mechanisms that confer high resistance to abiotic stressors such as elevated temperatures, water scarcity, salinity and intense solar or ultraviolet radiation. For this reason, they are also considered valuable bioindicators for assessing ecosystem responses to climate change (Novakovskaya et al. 2022; Penna et al. 2025). Many cyanobacteria can tolerate large temperature fluctuations and periods of freezing or drought by activating protective physiological mechanisms. These organisms are able to survive prolonged desiccation and rapidly resume metabolic activity after rehydration (Sharma et al. 2025). In addition, many cyanobacteria produce desiccation‐protective compounds, including polyols and extracellular polymeric substances (EPS), which help reduce water loss and improve water‐use efficiency (Samolov et al. 2020; Addesso et al. 2023). Under conditions of high irradiance, salinity or nutrient limitation, some species undergo encystment, during which they lose motility structures and form resistant cysts accompanied by mucilage secretion. This process is associated with the synthesis of photoprotective pigments (chlorophylls, carotenoids and phycobilins) and secondary metabolites, including polyphenols with antioxidant properties that protect cellular membranes and other sensitive cellular compartments from oxidative damage (Nandagopal et al. 2021).

In light of these characteristics, the potential biofertiliser, biostimulant and biopesticide functions of soil cyanobacteria and cyanobacteria‐enriched microbial communities have attracted increasing scientific interest. However, further research is required to elucidate their ecological roles and to support their practical application in agricultural systems, as their beneficial effects remain insufficiently documented in the literature. Accordingly, this study aimed to isolate, observe and characterise cyanobacteria‐dominated, nitrogen‐fixing microbial assemblages from the surface soils of a Mediterranean olive orchard in a semi‐arid climate that had been subjected to either sustainable or conventional agricultural management for 22 years. We hypothesise that cyanobacteria‐enriched microbial communities make a substantial contribution to soil quality and resilience under sustainable management, particularly in the current global context, where climate change and intensive farming practices are placing increasing pressure on agricultural soils, accelerating degradation processes and promoting desertification on a global scale.

Here, the term enrichment refers exclusively to a post‐sampling laboratory cultivation step designed to increase the relative abundance of cultivable cyanobacteria and associated nitrogen‐fixing bacteria for analytical purposes and does not imply any in situ soil amendment or manipulation.

## 2 Materials and Methods

### 2.1 Experimental Site and Agricultural Management

The study was conducted in a 2‐ha mature olive orchard ( *Olea europaea* L., cv. Maiatica), located in Ferrandina (Basilicata region, Southern Italy; 40°29′ N, 16°28′ E). Olive trees were approximately 70 years old, trained to a vase shape, planted at 8 × 8 m spacing and oriented northeast. The area is characterised by a semi‐arid Mediterranean climate, with a mean annual precipitation of 645 mm and an average annual temperature ranging between 15°C and 17°C (2020). The soil is classified as a sandy loam Haplic Calcisol, with low gravel content and increasing concentrations of finely divided calcium carbonate from the surface horizons (0–0.5 m) to the parent material (> 0.6 m). The site is located on a plain landform with a convex–straight slope and a gentle slope gradient (2%–5%). At the time of sampling, groundwater depth exceeded 1.5 m. Two adjacent plots (1 ha each), characterised by similar soil and tree properties, have been subjected to long‐term management systems since 2000.

#### 2.1.1 Sustainable Management (*S*_mng_)

The sustainably managed plot was conducted under organic agricultural practices. Olive trees were drip‐irrigated from March to October, with a total annual water supply of approximately 2800 m^3^ ha^−1^ year^−1^. Each tree was equipped with six drip emitters (8 L h^−1^) distributed within a 1‐m radius around the trunk. The top 60 cm of soil showed an average pH of 7.62 ± 0.36, total organic carbon of 10.82 ± 0.58 g kg^−1^, total nitrogen of 1.48 ± 0.28 g kg^−1^ and a C/N ratio of 6.99 ± 1.19, with a mean bulk density of 1.37 t m^−3^. Nutrient inputs supplied through irrigation water corresponded annually to 124 kg C, 54 kg N, 30 kg P and 50 kg K ha^−1^. An additional 40 kg ha^−1^ year^−1^ of nitrate nitrogen was applied via fertigation during fruit set and pit hardening to fully meet crop nutritional requirements. Trees were lightly pruned annually during winter. The soil surface was permanently covered by spontaneous vegetation (mainly Fabaceae and Poaceae), mowed twice per year. Pruning residues and cover crop biomass were shredded and left on the soil surface as mulch.

#### 2.1.2 Conventional Management (*C*_mng_)

The conventionally managed plot followed local farming practices under rainfed conditions. The top 60 cm of soil had an average pH of 7.97 ± 0.31, organic carbon content of 9.78 ± 0.20 g kg^−1^, total nitrogen of 1.05 ± 0.12 g kg^−1^ and a C/N ratio of 9.32 ± 1.40, with a mean bulk density of 1.22 t m^−3^. Weed control was performed through mechanical tillage (milling at approximately 10 cm depth) two to three times per year. Severe pruning was carried out every 2 years, and pruning residues were removed from the orchard. Mineral fertilisation was performed once per year in early spring using NPK (20–10–10) fertilisers at rates ranging from 300 to 500 kg ha^−1^ year^−1^.

No symptoms of nutrient deficiencies, diseases or severe abiotic stress were observed in either management system. Tree height (approximately 4.0–4.5 m) and trunk diameter were comparable between treatments.

### 2.2 Soil Sampling

Soil sampling was performed in February 2022. For each management system (*S*_mng_ and *C*_mng_), three independent composite soil samples were collected (*n* = 3), considering the 1‐ha extension of each plot. To avoid edge effects and potential treatment interference, border areas were excluded from sampling. Using sterile gloves and spatulas, the upper 2–3 cm of soil were collected from multiple microsites targeting edaphic cyanobacteria, including areas close to tree trunks, trunk cavities, along and between rows, under canopies and open spaces, and both near and distant from drip emitters. A total of 50 subsamples were collected per composite sample, pooled into sterile plastic bags to obtain approximately 500 g of soil. Composite sampling was repeated three times for each management system. Samples were stored at room temperature and processed for cyanobacterial enumeration within 24 h.

### 2.3 Enumeration of Soil Cyanobacteria

Soil cyanobacteria were quantified using the most probable number (MPN) technique. Soil suspensions were prepared by adding 10 g of fresh soil to 90 mL of distilled water containing 0.1 g sodium pyrophosphate, followed by shaking at 100 rpm for 10 min to obtain a 10^−1^ (w/v) suspension. Serial dilutions (10^−2^, 10^−3^ and 10^−4^) were prepared accordingly.

A modified Bristol liquid medium devoid of nitrogen was used to selectively promote the growth of nitrogen‐fixing cyanobacteria and associated diazotrophic microorganisms. The absence of inorganic nitrogen sources imposes a selective pressure favouring organisms capable of atmospheric N_2_ fixation, thereby reducing the proliferation of non‐diazotrophic heterotrophs during cultivation. The medium contained (g L^−1^): CaCl_2_ (0.025), MgSO_4_·7H_2_O (0.075), K_2_HPO_4_ (0.075), KH_2_PO_4_ (0.018), NaCl (0.025) and FeCl_3_ (0.005). For each dilution (10^−2^ to 10^−4^), 1 mL of soil suspension was inoculated into 9 mL of medium, and 10 replicate tubes were prepared per dilution and treatment.

Cultures were incubated for 30 days under controlled conditions (25°C; 12 h light/12 h dark photoperiod) using full‐spectrum LED lighting (18 W). Tubes were periodically inspected for cyanobacterial growth. After incubation, the number of positive tubes at each dilution was recorded, and cyanobacterial abundance was estimated using MPN tables, expressed as cells g^−1^ fresh soil.

### 2.4 Microscopic Observation and Morphological Characterisation of Cyanobacteria

For each composite soil sample, three tubes exhibiting clear cyanobacterial growth (typically from the 10^−2^ dilution) were selected for microscopic analysis. Cultures were homogenised by gentle shaking, and aliquots were mounted on glass slides, covered with coverslips and immediately observed using a light microscope (ADL 601 P, Bresser GmbH, Germany) equipped with a digital camera (MikroCam II 20 MP). Observations were performed under transmitted and polarised light, using phase contrast, at ×400 and ×600 magnifications. For each tube, three slides were prepared, resulting in nine observations per soil sample and 27 observations per treatment.

### 2.5 DNA Extraction, 16S rRNA Gene Library Preparation, Sequencing and Bioinformatics Analyses

Bacterial community composition was investigated using DNA extracted from cyanobacteria‐enriched microbial assemblages obtained under nitrogen‐free culture conditions. Three independent biological replicates per management system (*n* = 3), corresponding to the three composite soil samples described above, were processed for DNA extraction, library preparation, sequencing and downstream bioinformatic analyses. Enriched biomass was harvested from liquid cultures, and total DNA was extracted using the FastDNA SPIN Kit for Soil (MP Biomedicals, USA). Samples were processed with Lysing Matrix E and subjected to mechanical homogenisation using a FastPrep‐24 5G instrument (40 s at 6.0 m s^−1^). After centrifugation, proteins were precipitated and DNA was selectively bound to a silica‐based binding matrix, washed and eluted in 100 μL of elution buffer. DNA concentration was determined fluorometrically using the Qubit Flex Fluorometer (Invitrogen, Carlsbad, CA, USA). DNA integrity and fragment size distribution were evaluated using the Agilent 4200 TapeStation system with the Genomic DNA ScreenTape kit (Agilent Technologies, Santa Clara, CA, USA), which provided DNA Integrity Number (DIN) values for quality assessment.

For bacterial community profiling, sequencing libraries targeting the V3–V4 hypervariable regions of the 16S rRNA gene were prepared according to the Illumina 16S Metagenomic Sequencing Library Preparation protocol. Amplicon PCR was performed using region‐specific primers containing Illumina overhang adapters and KAPA HiFi HotStart ReadyMix. Amplification conditions consisted of an initial denaturation at 95°C, followed by cycles of denaturation, annealing and extension, and a final extension step. PCR products were purified using Agencourt AMPure XP magnetic beads to remove primers and non‐specific fragments.

Indexing PCR was subsequently performed to attach dual i5 and i7 indices and Illumina sequencing adapters using Nextera XT index primers. Indexed libraries were purified again with AMPure XP beads to eliminate adapter dimers and short fragments. Final library quality and fragment size distribution were verified using the Agilent 4200 TapeStation with the D1000 assay. Libraries were quantified using the Qubit Flex Fluorometer, normalised to 4 nM, and pooled equimolarly. The pooled library was denatured, diluted and combined with a 30% PhiX Control Library (v3) to improve sequence diversity and monitor sequencing performance. Sequencing was carried out on an Illumina MiSeq platform using 2 × 250 bp paired‐end chemistry.

Bioinformatics processing was conducted using the QIIME2 pipeline (Bolyen et al. 2019). Primer sequences and Illumina adapters were removed with Cutadapt, and read quality was assessed using FastQC and MultiQC. Paired‐end reads were denoised using the Deblur plugin to generate amplicon sequence variants (ASVs) (Amir et al. 2017). Taxonomic assignment was performed using a V3–V4‐specific classifier trained on the SILVA reference database (release 138) (Kaehler 2022). Alpha diversity was quantified from 16S rRNA gene amplicon sequences using Faith's phylogenetic diversity (Faith's PD) index calculated on ASVs generated in QIIME2 (v. 2025.10). Differences in alpha diversity between management systems were evaluated using the Mann–Whitney *U* test. Beta diversity was assessed using Bray–Curtis dissimilarity matrices calculated from relative abundance data, and differences in community composition were tested by PERMANOVA with 999 permutations. After quality filtering, denoising and chimaera removal, a total of 227,061 high‐quality sequences were retained across samples, resulting in 645 ASVs for downstream diversity and taxonomic analyses.

Partial least squares discriminant analysis (PLS–DA) was performed on relative abundance data to visualise differences in bacterial community composition between management systems and to identify the taxa contributing most strongly to sample discrimination. To complement the multivariate analysis, fold‐change (FC) analysis was conducted to quantify the magnitude and direction of differences in taxon abundance between treatments. Phyla with FC values > 2 or < 0.5 were considered differentially enriched between management systems.

### 2.6 Untargeted Metabolomics Analysis of Cyanobacteria

Untargeted metabolomic profiling of cyanobacterial cells and culture supernatants was performed using ultra‐high‐performance liquid chromatography coupled to quadrupole time‐of‐flight mass spectrometry (UHPLC/QTOF‐MS; Agilent Technologies, USA). For metabolomic analyses, five independent biological replicates were analysed for each management system and sample fraction (cell extracts and culture supernatants; *n* = 5 per treatment). Cyanobacterial cultures were centrifuged at 5000*g* for 30 min to separate cells from supernatant. Supernatants were filtered through 0.22‐μm cellulose syringe filters before analysis. Cell pellets were extracted using 80% methanol containing 0.1% formic acid (v/v) and subjected to ultrasound‐assisted extraction (120 W, 15 min). A volume of 19 μL of each extract or supernatant was injected into the UHPLC/QTOF‐MS system. Separation was achieved using a reverse‐phase C18 column (Agilent Zorbax, 15 cm × 2.1 mm, 1.7 μm particle size) with a water–acetonitrile gradient (6%–94% acetonitrile over 33 min). Mass spectra were acquired in positive ionisation mode over an *m*/*z* range of 100–1200, with a nominal resolution of 30,000 FWHM.

### 2.7 Metabolite Annotation and Statistical Analysis

Raw mass spectrometry data were processed using Agilent Profinder B.07 software, applying the ‘find‐by‐formula’ algorithm against a dedicated cyanobacterial metabolite database. Metabolite annotation followed the COSMOS Metabolomics Standards Initiative guidelines (Level 2), using a mass accuracy tolerance of 5 ppm. Features were retained if present in at least 75% of replicates within at least one experimental group.

Multivariate analyses, including hierarchical cluster analysis (HCA) and principal component analysis (PCA), were performed using Mass Profiler Professional B12.6 (Agilent Technologies). Supernatant metabolites showing statistically significant differences between *S*_mng_ and *C*_mng_ treatments (*p* < 0.05, Benjamini–Hochberg corrected) were subjected to chemical class enrichment analysis using ChemRICH. Supervised orthogonal partial least squares discriminant analysis (OPLS–DA) was conducted using MetaboAnalyst 5.0, with model validation based on 100 permutation tests. Metabolites with high variable importance in projection (VIP) scores were identified. To assess the statistical significance and robustness of the OPLS‐DA model, a cross‐validated analysis of variance (CV‐ANOVA) was performed on the metabolomic dataset. Model significance was evaluated using the CV‐ANOVA F statistic and the associated *p*‐value, with *p* < 0.05 considered statistically significant. Moreover, pathway analysis for cyanobacterial cell metabolites was performed using MetaCyc Pathway Tools, considering statistically significant metabolites (*p* < 0.05) with a fold change > 2.

## 3 Results

### 3.1 Soil Cyanobacterial Abundance and Microscopic Observations

Quantification of soil cyanobacteria showed a significant effect of long‐term soil management. The abundance of cultivable nitrogen‐fixing cyanobacteria enriched under nitrogen‐free conditions was significantly higher in soils under sustainable management (*S*_mng_; 0.408 × 10^2^ cells g^−1^ fresh soil) than in conventionally managed soils (*C*_mng_; 0.240 × 10^2^ cells g^−1^ fresh soil) (Student's *t*‐test, *t* = 7.01, *p* = 0.0022).

**FIGURE 1:**
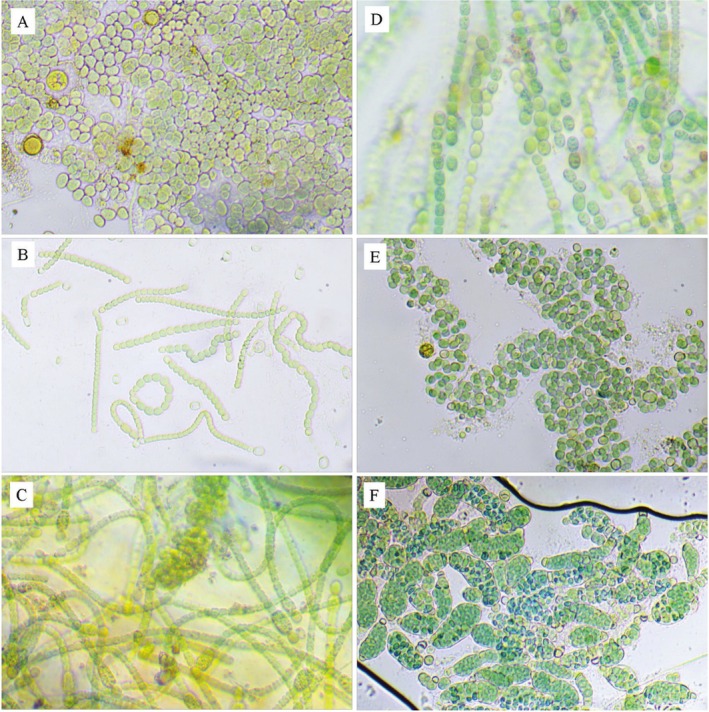
Representative cyanobacterial morphotypes observed in cyanobacteria‐enriched cultures derived from sustainably (*S*_mng_) and conventionally (*C*_mng_) managed soils. *S*_mng_ samples show structurally complex morphotypes, including coccoid cyanobacteria forming compact colonies (Chroococcales‐like; A), thin non‐heterocystous filamentous cyanobacteria (*Leptolyngbya*‐like; B) and dense filamentous networks (*Phormidium*/*Leptolyngbya*‐like; C). *C*_mng_ samples display simpler and disturbance‐tolerant morphologies, including loosely aggregated coccoid cyanobacteria (Chroococcales‐like; D), very thin isolated filamentous trichomes (*Leptolyngbya*‐like; E) and fragmented filamentous forms with hormogonia‐like structures (*Phormidium*‐like; F). Images were obtained under transmitted light and phase‐contrast microscopy from nitrogen‐free enrichment cultures.

Microscopic observations of cyanobacteria‐enriched cultures derived from *S*_mng_ revealed a marked morphological diversity dominated by coccoid and filamentous cyanobacteria. Light and phase‐contrast microscopy showed abundant coccoid forms consistent with Chroococcales‐like morphotypes (e.g., *Chroococcus*/*Gloeocapsa*‐like), occurring as single cells or small colonies embedded in mucilaginous matrices (Figure 1A). In addition, non‐heterocystous filamentous cyanobacteria belonging to Oscillatoriales‐like morphotypes were prevalent, including thin trichomes attributable to *Leptolyngbya*‐like taxa (Figure 1B) and thicker filaments forming dense, intertwined networks resembling *Phormidium*/*Leptolyngbya*‐like morphologies (Figure 1C). These observations indicate that *S*_mng_ soils support structurally diverse cyanobacterial assemblages capable of forming both unicellular and filamentous growth forms under nitrogen‐free enrichment conditions.

In contrast, cyanobacteria‐enriched cultures derived from *C*_mng_ showed a less complex morphological organisation. Microscopic observations revealed coccoid cyanobacteria forming loose aggregates with limited mucilaginous development, consistent with Chroococcales‐like morphotypes (Figure 1D). Filamentous forms were dominated by very thin, non‐heterocystous Oscillatoriales‐like trichomes attributable to *Leptolyngbya*‐like taxa (Figure 1E), often occurring as isolated or weakly interconnected filaments. In addition, fragmented filamentous morphotypes displaying short trichome segments and hormogonia‐like structures were frequently observed, resembling *Phormidium*‐like cyanobacteria (Figure 1F).

### 3.2 Community Structure of Cyanobacteria‐Enriched Microbial Assemblages From Metabarcoding

**FIGURE 2:**
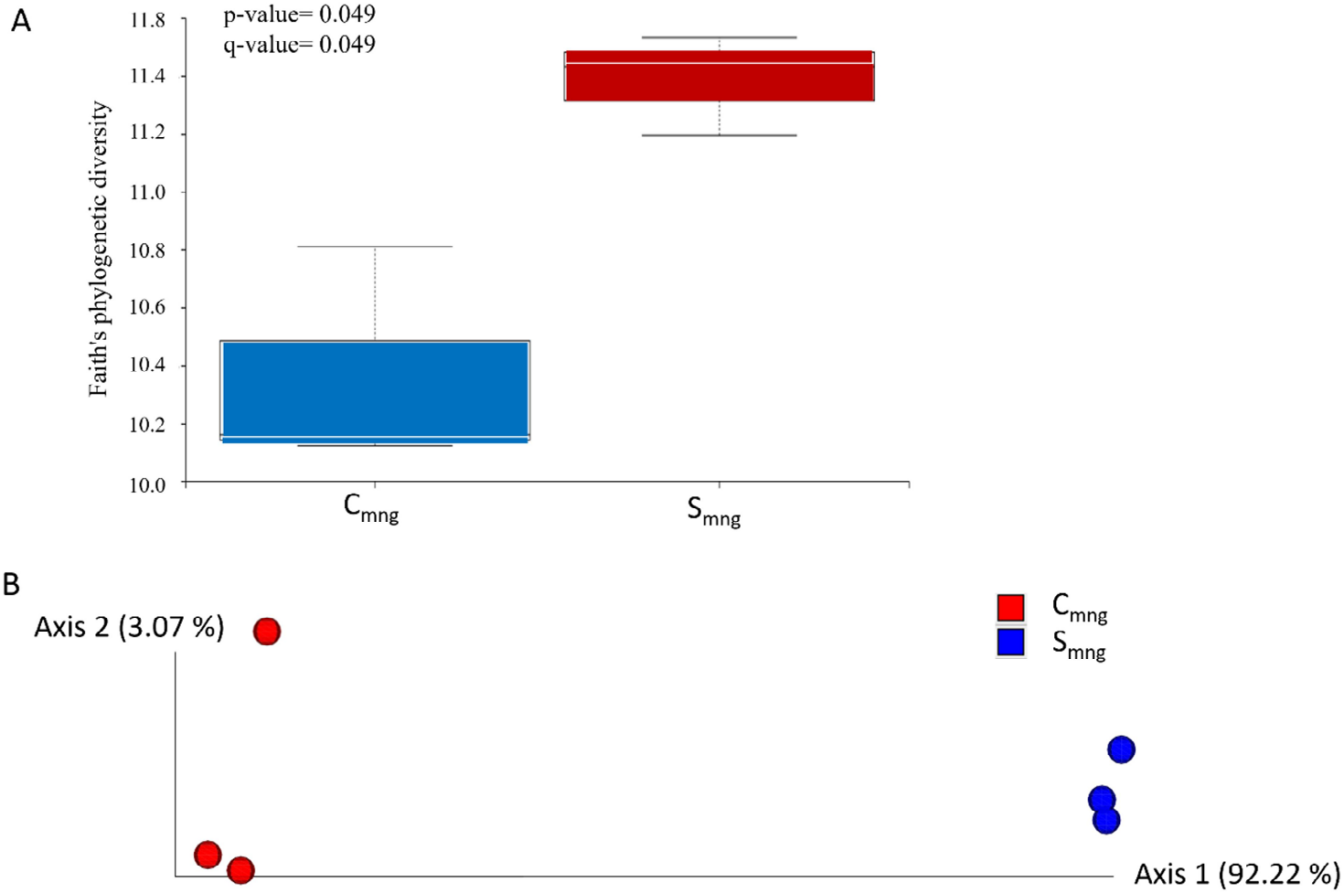
Alpha and beta diversity of bacterial communities under different soil management systems. (A) Alpha diversity expressed as Faith's phylogenetic diversity (Faith's PD), with statistical differences assessed using a Kruskal–Wallis pairwise test. (B) Beta diversity based on Bray–Curtis dissimilarity, showing differences in community composition between sustainable (*S*_mng_) and conventional (*C*_mng_) soil management.

Alpha diversity analysis revealed differences in the phylogenetic diversity of bacterial communities enriched under nitrogen‐free conditions between *S*_mng_ and *C*_mng_ systems, as indicated by Faith's PD values (Figure 2A). Beta diversity analysis, using Bray–Curtis dissimilarity, showed a clear separation between samples from the two management types, indicating distinct cyanobacteria‐dominated bacterial community compositions within the enriched assemblages (Figure 2B).

**FIGURE 3:**
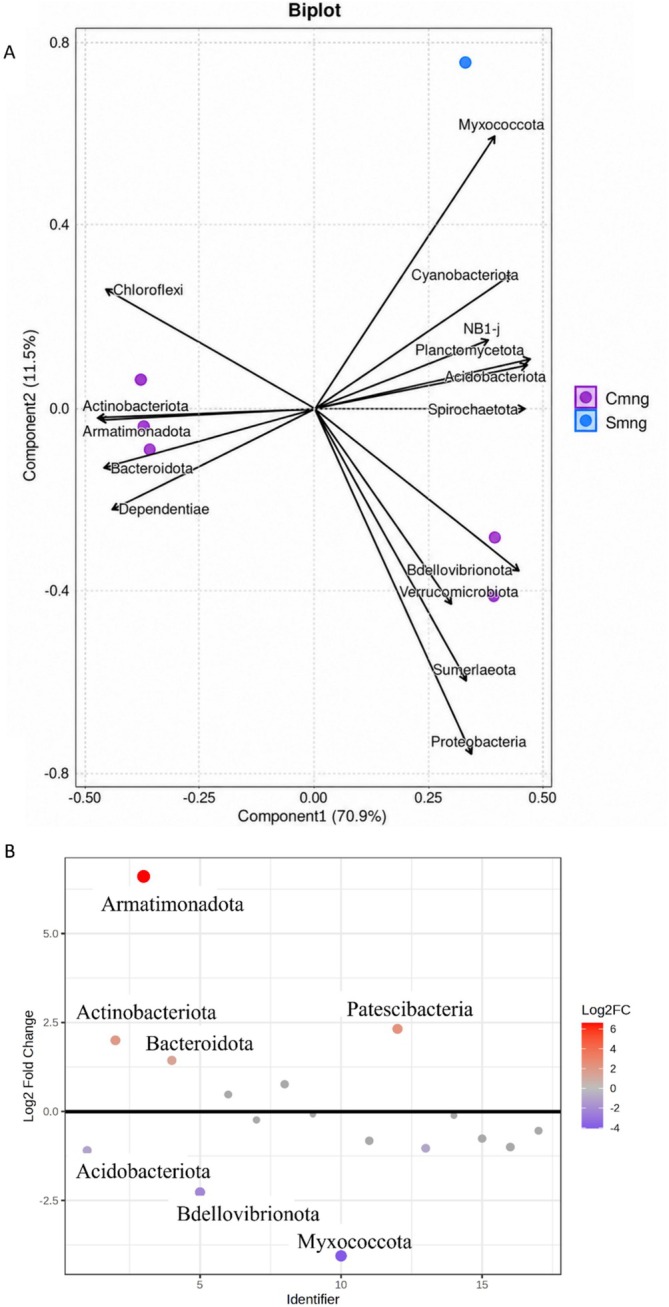
Partial least squares discriminant analysis (PLS–DA) biplot (A) illustrating differences in bacterial communities enriched under nitrogen‐free conditions between soils under sustainable management (*S*_mng_) and conventional management (*C*_mng_). Component 1 explains 70.9% of the variance, and Component 2 explains 15.5% of the variance. Vectors indicate the contribution of bacterial phyla. *S*_mng_ samples are shown in green and *C*_mng_ samples in red. Fold change (FC) analysis (B) comparing bacterial phyla abundances between conventionally managed (*C*_mng_) and sustainably managed (*S*_mng_) soils. FC values were calculated on normalised data as the ratio between group means (*C*_mng_/*S*_mng_). Phyla with FC > 2 or FC < 0.5 were considered significantly up‐ or down‐regulated, respectively. Positive FC values indicate higher phyla abundance under *C*_mng_, whereas negative FC values indicate higher abundance under *S*_mng_.

**FIGURE 4:**
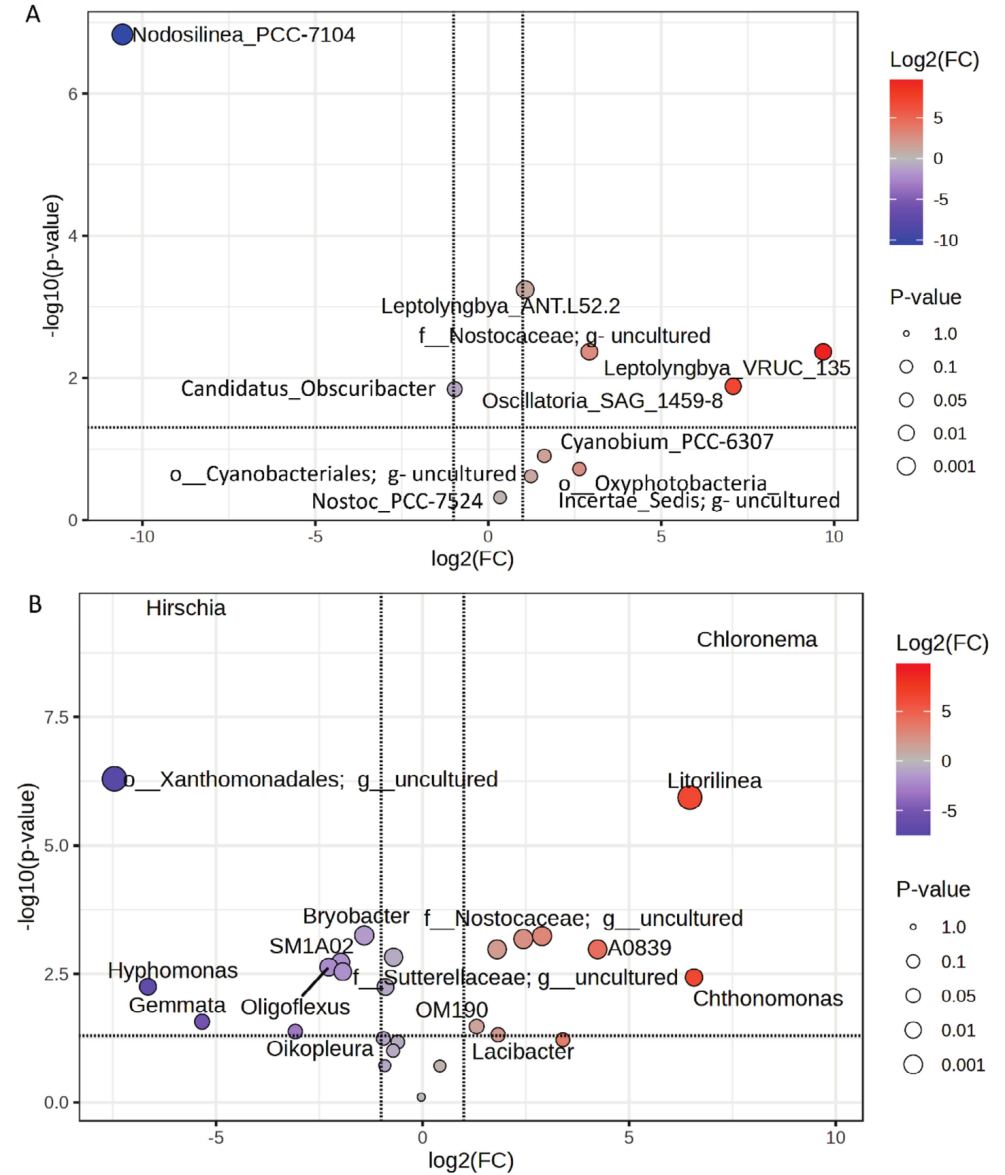
Volcano plots showing differential taxonomic abundance within cyanobacteria‐enriched microbial assemblages recovered from conventionally managed (*C*_mng_) and sustainably managed (*S*_mng_) soils. (A) Cyanobacterial genera with a mean relative abundance > 0.1% in at least one treatment. (B) Non‐cyanobacterial genera with a mean relative abundance > 1% in at least one treatment. Volcano plots were generated using log_2_ fold‐change (FC) analysis combined with Welch's *t*‐test (raw *p* < 0.05; FC threshold = 2). Positive log_2_FC values indicate taxa enriched in *C*_mng_ soils, whereas negative log_2_FC values indicate taxa enriched in *S*_mng_ soils. Point size reflects statistical significance (−log_10_ *p*‐value), and colour intensity represents the magnitude and direction of fold change.

The PLS–DA analysis revealed clear separation between bacterial communities enriched under nitrogen‐free conditions in *S*_mng_ and *C*_mng_ soils, primarily along Component 1, which explained 70.9% of the total variance (Figure 3A). This result indicates that soil management was the principal factor shaping the composition of the cyanobacteria‐enriched bacterial assemblages. *S*_mng_ samples clustered on the positive side of Component 1, whereas *C*_mng_ samples clustered on the negative side. The loading plot suggested that Cyanobacteria, Proteobacteria, Acidobacteriota, Verrucomicrobiota, Gemmatimonadota, Firmicutes, Nitrospirota and Myxococcota contributed positively to the separation of *S*_mng_ samples. In contrast, Actinobacteriota, Chloroflexi, Bacteroidota, Armatimonadota and Dependentiae were more closely associated with *C*_mng_ samples. To further resolve the taxa underlying these differences, differential abundance analyses were subsequently performed at the genus level for both cyanobacterial and non‐cyanobacterial members of the enriched assemblages (Figure 4).

Moreover, fold change analysis revealed pronounced differences in the bacterial communities enriched under nitrogen‐free conditions at the phylum level between *C*_mng_ and *S*_mng_ systems (Figure 3B). Using a fold change threshold of ±2, several phyla showed significant differential abundance between *C*_mng_ and *S*_mng_ conditions. A subset of features showed FC values greater than 2 (e.g., Armatimonadota, Actinobacteriota and Bacteroidota), indicating higher relative abundance in bacterial phyla from *C*_mng_, while other phyla displayed FC values below 0.5 (e.g., Myxococcota, Bdellovibrionota and Acidobacteriota), corresponding to higher abundance under *S*_mng_. The asymmetric distribution of fold changes indicates that soil management strongly influenced both the direction and magnitude of phyla responses.

Differential abundance analysis based on log_2_ fold change and Welch's *t*‐test identified several cyanobacterial genera within the cyanobacteria‐enriched microbial assemblages that differed significantly between the two soil management systems (Figure 4A). Genera with positive log_2_(FC) values were more abundant under conventional management (*C*_mng_), whereas genera with negative log_2_(FC) values were enriched under sustainable management (*S*_mng_). The combined evaluation of fold change and statistical significance highlighted a subset of dominant cyanobacterial genera (≥ 0.1%) strongly associated with a specific management regime, indicating a clear management‐dependent structuring of cyanobacteria‐enriched communities recovered under selective culture conditions.

In conventionally managed soils, the filamentous genus *Leptolyngbya* was dominant, accounting for approximately 38%–43% of the total cyanobacterial community across *C*_mng_ samples. A complete overview of the relative abundances of cyanobacterial taxa in the enriched assemblages is provided in Figure S1. Minor contributions were also observed for *Leptolyngbya* VRUC_135 (≈1.5%–2.7%), *Nostoc* (≈0.7%–1.7%) and *Oscillatoria* (≈0.3%–0.7%). In contrast, sustainably managed soils were characterised by a marked increase in *Nodosilinea*, which represented approximately 26%–27% of the cyanobacterial community, while *Leptolyngbya* abundance decreased to 17%–22%.

Analysis of non‐cyanobacterial genera (≥ 1%) associated with the cyanobacteria‐enriched assemblages also revealed clear management‐dependent differences (Figure 4B). Genera with positive log_2_(FC) values, including *Chloronema*, *Litorilinea*, *Chthonomonas* and *A0839*, were more abundant in *C*_mng_‐derived assemblages, whereas *Hirschia*, *Hyphomonas*, *Gemmata*, *Oligoflexus* and an uncultured member of the *Xanthomonadales* were enriched in *S*_mng_‐derived assemblages. Several additional taxa, including *Bryobacter*, *SM1A02* and uncultured members of the *Nostocaceae* and *Sutterellaceae*, showed intermediate but significant shifts between management systems. These results indicate that the selective enrichment conditions influenced not only cyanobacterial composition but also the structure of the associated heterotrophic bacterial community.

### 3.3 Cyanobacterial Metabolomic Profiles Under Contrasting Soil Managements

**FIGURE 5:**
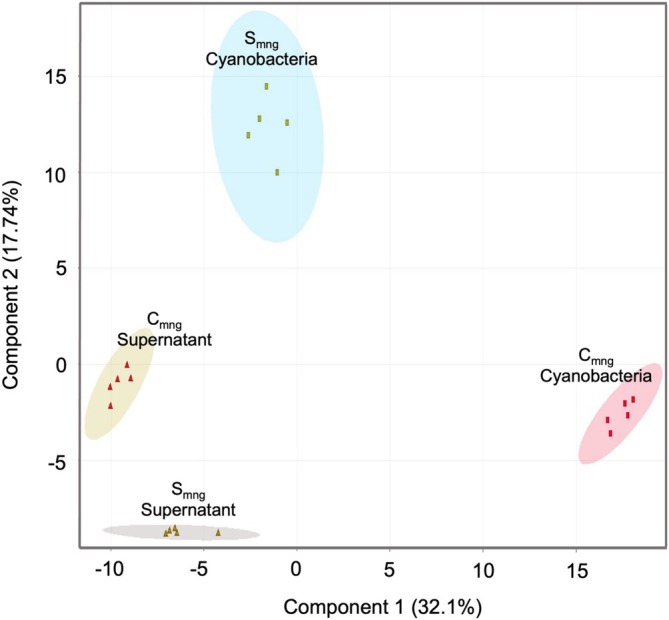
Principal component analysis (PCA) of metabolomic profiles of cyanobacteria‐enriched microbial assemblages derived from sustainable (*S*_mng_) and conventional (*C*_mng_) soil management systems. The term ‘Cyanobacteria’ denotes metabolite profiles obtained from methanolic extracts of cyanobacterial cell pellets, whereas ‘Supernatant’ denotes extracellular metabolite profiles obtained from the corresponding cell‐free culture supernatants.

Untargeted metabolomic profiling of cyanobacteria‐enriched cultures and their extracellular metabolites revealed pronounced differences between *S*_mng_ and *C*_mng_ soils. A total of 494 metabolic features were detected across all cyanobacteria‐associated enriched samples. Unsupervised PCA highlighted a clear separation between cyanobacteria‐enriched samples derived from *S*_mng_ and *C*_mng_ soils (Figure 5). The first two principal components explained 49.84% of the total variance, with soil management being the main discriminating factor along PC2. This result indicates a strong influence of agricultural practices on the metabolic activities of cyanobacteria‐dominated, nitrogen‐fixing microbial assemblages recovered under selective culture conditions.

HCA further supported the PCA results, revealing distinct clustering of *S*_mng_ and *C*_mng_ samples based on metabolite abundance patterns (Figure S2). Cyanobacteria‐enriched assemblages isolated from *S*_mng_ soils displayed specific up‐ and down‐regulation of several metabolites that were absent or weakly expressed in *C*_mng_ isolates.

### 3.4 Cyanobacterial Metabolic Pathways Modulated by Soil Management

**FIGURE 6:**
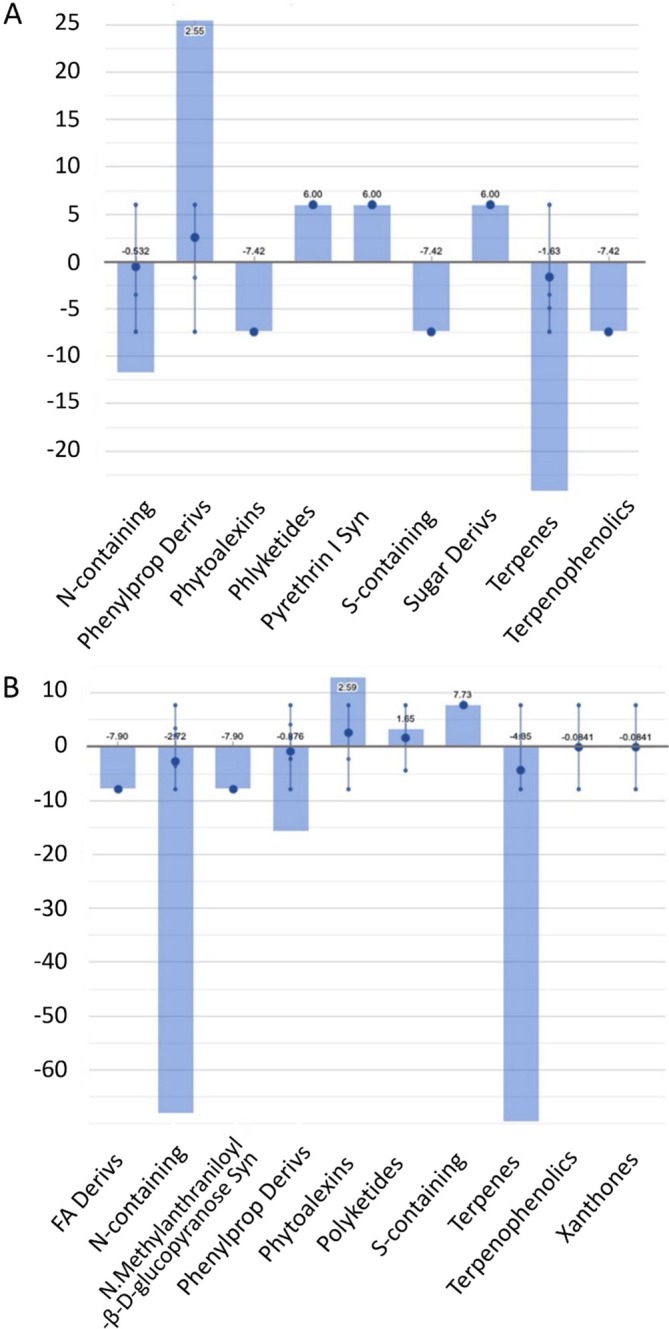
Differentially modulated metabolite classes detected in culture supernatants and cyanobacterial cell extracts from cyanobacteria‐enriched microbial assemblages under sustainable (*S*_mng_) and conventional (*C*_mng_) soil management. The combined overview highlights differential modulation of chemical classes across (A) extracellular and (B) intracellular fractions, illustrating compartment‐specific metabolic responses. Positive values indicate metabolite classes enriched in *S*_mng_‐derived assemblages relative to *C*_mng_, whereas negative values indicate metabolite classes enriched in *C*_mng_‐derived assemblages relative to *S*_mng_. The cellular fraction was subsequently analysed in detail using MetaCyc pathway analysis (Figure 7) to resolve management‐driven shifts in intracellular biosynthetic, amino acid and signalling pathways.

The integrated MetaCyc output for metabolite biosynthesis revealed that long‐term soil management modulated distinct chemical classes differently in the supernatant (Figure 6A) and the cell extract (Figure 6B). In the supernatant, the strongest class‐level contrasts involved phenylpropanoid derivatives, terpenes, terpenophenolics, phytoalexins, polyketides, pyridoxine, sugar derivatives and N‐ and S‐containing compounds. Several of these classes showed marked differences between sustainable and conventional management, indicating that long‐term management influenced the composition of extracellular metabolites across multiple chemical groups. In the cell fraction, a partially distinct set of dominant classes contributed to the observed separation, with prominent modulation of fatty acid derivatives, terpenes/terpenophenolics and S‐containing compounds (Figure 6).

**FIGURE 7:**
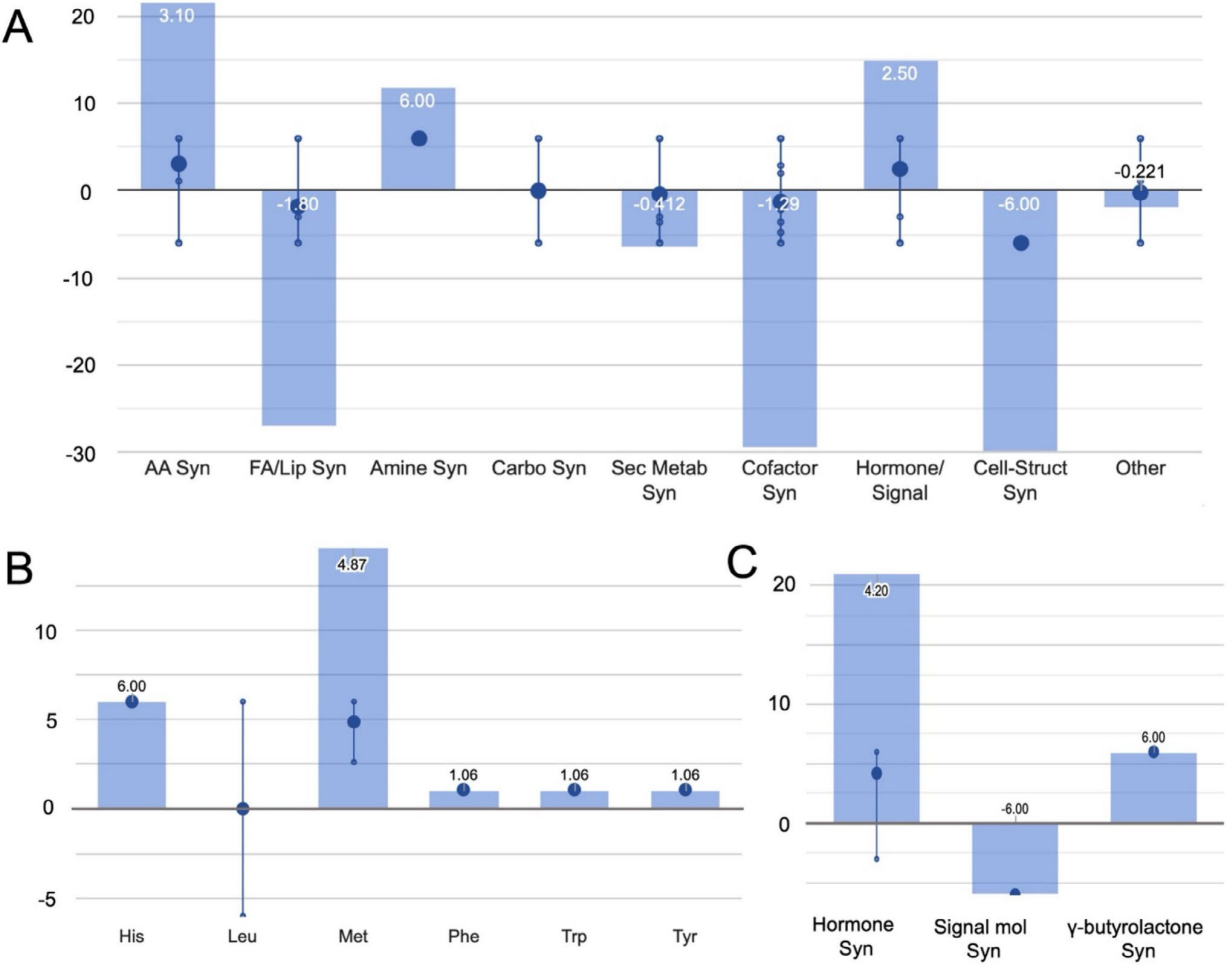
Differentially modulated metabolic pathways in cyanobacteria‐enriched microbial assemblages under sustainable (*S*_mng_) compared to conventional (*C*_mng_) management soils. (A) Biosynthetic pathways; (B) amino acid biosynthesis and (C) hormone and signalling molecule biosynthesis pathways. Positive log fold‐change values indicate pathways enriched in *S*_mng_‐derived assemblages, whereas negative values indicate pathways enriched in *C*_mng_‐derived assemblages. Large dots represent average log fold‐change values for each metabolic class, while small dots represent individual metabolites or pathways contributing to each category. AA, amino acids; Amine, amines and polyamines; Carbo, carbohydrates; Cell‐struct, plant cell structures; Cofactors, cofactors, prosthetic groups, electron carriers, and vitamins; FA/lipids, fatty acids and lipids; His, histidine; Hormone/Signal, Hormone, Neurotransmitter and Signalling Molecule Biosynthesis; Leu, Leucine; Met, methionine; Nucleo, nucleosides and nucleotides; Phe, phenylalanine; Sec metab, secondary metabolism; Signal mol, signal molecules.; Trp, tryptophane; Tyr, tyrosine.

Focusing on the cellular fraction, pathway‐level analysis using MetaCyc provided a detailed view of intracellular metabolic reprogramming under *S*_mng_ and *C*_mng_ systems (Figure 7). Cultivation‐derived cyanobacteria‐dominated microbial communities from *S*_mng_ soils showed an overall down‐regulation of multiple biosynthetic pathways, particularly those related to FA and lipid biosynthesis, cofactors and vitamin synthesis, secondary metabolism and cell structural components (Figure 7A). This pattern indicates reduced investment in energy‐ and resource‐intensive biosynthetic processes under *S*_mng_ conditions.

In contrast, assemblages derived from *C*_mng_ soils stimulated the biosynthesis of FAs, especially branched, hydroxylated, epoxidised and unsaturated FAs, as well as phospholipids, cutin‐related compounds, eicosanoids and S‐containing lipids. Vitamin biosynthesis pathways, including phylloquinone, thiamine and vitamins A, B6, E and K, were also up‐regulated under *C*_mng_, whereas menaquinone‐related superpathways were preferentially enhanced in *S*_mng_‐derived enriched assemblages.

Moreover, *S*_mng_‐derived cyanobacteria‐enriched assemblages promoted the up‐regulation of amino acid biosynthesis pathways (Figure 7B) and hormone‐ and signalling‐related metabolites (Figure 7C). Among these, cytokinin biosynthesis pathways (including *cis*‐ and *trans*‐zeatin) and ethylene biosynthesis‐related pathways were significantly enriched, while leukotriene‐related pathways were down‐regulated, suggesting a potential role of cyanobacteria‐dominated microbial communities associated with *S*_mng_ soils in plant growth promotion and stress signalling.

**FIGURE 8:**
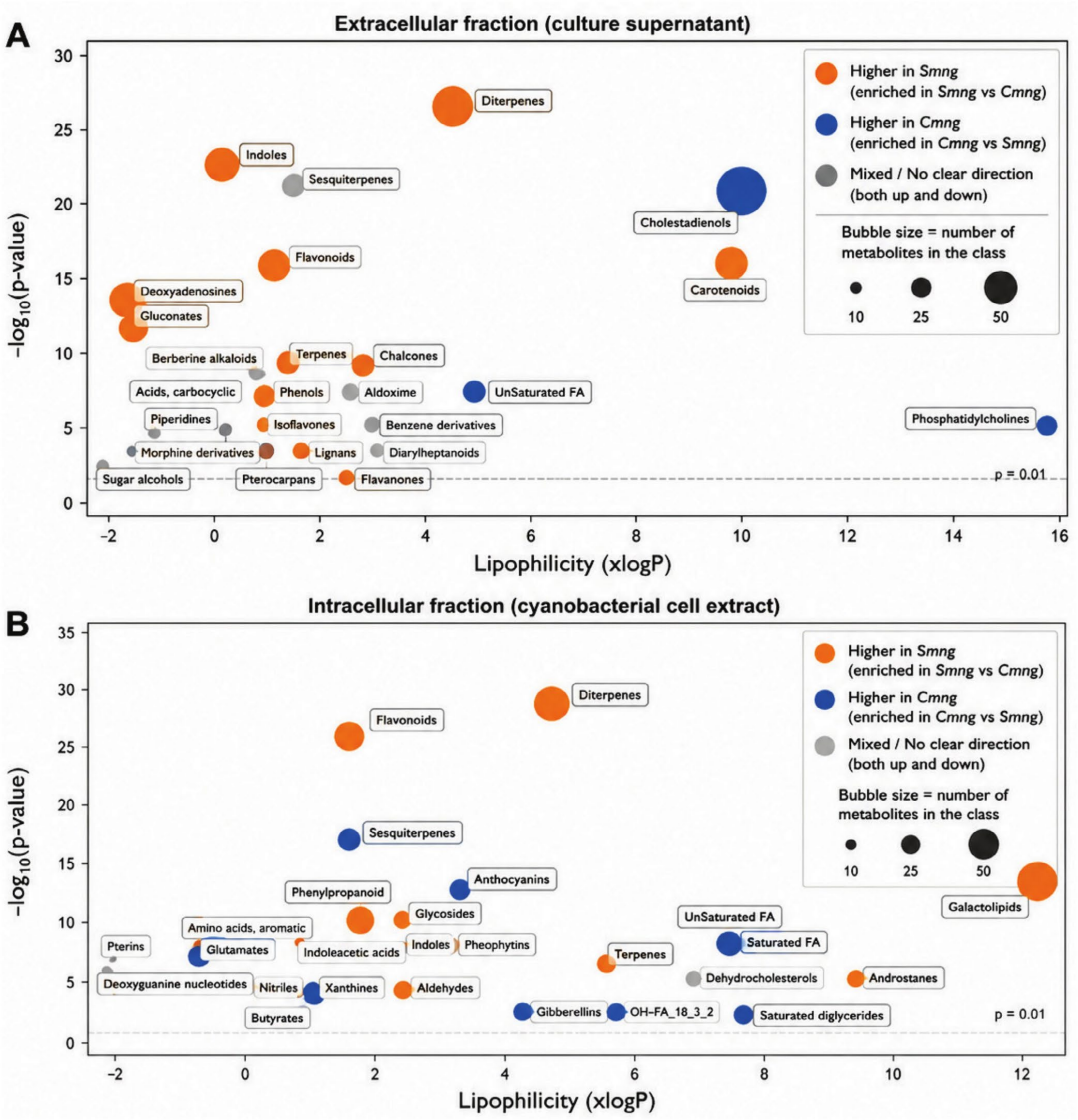
Chemical similarity enrichment analysis (ChemRICH) of metabolites significantly modulated between sustainable (*S*_mng_) and conventional (*C*_mng_) soil management systems after Benjamini–Hochberg correction (*q* < 0.05). (A) ChemRICH analysis of metabolites detected in culture supernatants of cyanobacteria‐enriched microbial assemblages, representing extracellularly released compounds. (B) ChemRICH analysis of metabolites detected in cyanobacterial cell extracts, representing the intracellular fraction. The *x*‐axis represents metabolite lipophilicity (*X*log *P*), whereas the *y*‐axis indicates statistical significance (−log_10_ *p*‐value). Bubble size is proportional to the number of metabolites within each chemical class. Orange bubbles indicate chemical classes enriched in *S*_mng_‐derived assemblages, blue bubbles indicate classes enriched in *C*_mng_‐derived assemblages and grey bubbles represent classes showing mixed accumulation patterns or no clear directional enrichment.

Furthermore, ChemRICH analysis revealed that long‐term soil management significantly altered the chemical composition of both extracellular and intracellular metabolite pools of cyanobacteria‐enriched microbial assemblages after Benjamini‐Hochberg correction (*q* < 0.05) (Figure 8). In the supernatant fraction (Figure 8A), management‐dependent modulation was observed across multiple metabolite classes associated with extracellular release. Several chemical clusters showed a predominance of compounds increased under *S*_mng_ conditions, while other classes were preferentially enriched under *C*_mng_. These clusters mainly comprised metabolites typically involved in cell–environment interactions, signalling processes and extracellular carbon and nitrogen exchange, whereas mixed clusters suggested a differential regulation of secretion pathways rather than uniform activation or repression. The pronounced restructuring of extracellular metabolite classes indicates that soil management strongly influenced the metabolic signals released by cyanobacteria‐enriched assemblages into their surrounding environment. In contrast, ChemRICH analysis of the intracellular metabolite pool (Figure 8B) revealed a distinct pattern of class‐level modulation, reflecting changes in internal metabolic organisation rather than secretion dynamics. Intracellular metabolite classes displayed clearer directional responses to management, consistent with shifts in biosynthetic investment, energy metabolism and cellular maintenance processes.

**FIGURE 9:**
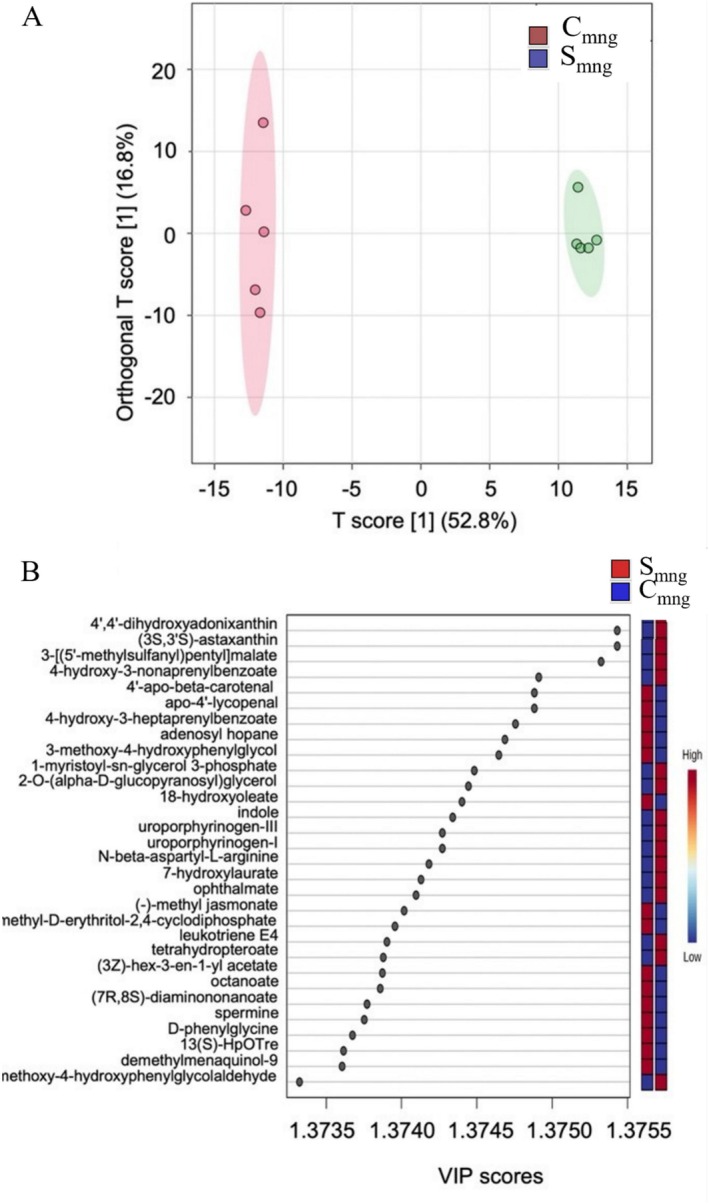
(A) Orthogonal projections to latent structures discriminant analysis (OPLS–DA) of metabolomic profiles of supernatants derived from cyanobacteria‐enriched microbial assemblages, discriminating sustainable (*S*_mng_) and conventional (*C*_mng_) soil management systems. (B) Variable importance in projection (VIP) scores of metabolites contributing most strongly to sample separation.

To discriminate management‐specific metabolic signatures, supervised orthogonal projections to latent structures discriminant analysis (OPLS–DA) was applied to supernatant metabolomic data from cyanobacteria‐enriched cultures (Figure 9A). The model showed excellent performance, with high goodness‐of‐fit (*R* ^2^ *Y* = 0.95) and predictability (*Q* ^2^ = 0.92). The first latent variable accounted for 52.8% of the total variance, clearly separating *S*_mng_ and *C*_mng_ samples. The statistical significance of the OPLS–DA model was further confirmed by CV‐ANOVA, which indicated a highly significant separation between treatments (*F* = 50.60, *p* = 1.0 × 10^−4^), supporting that the observed discrimination was unlikely to result from model overfitting. Variable importance in projection (VIP) analysis identified a subset of metabolites strongly contributing to this separation (Figure 9B). These metabolites are proposed as potential biomarkers of metabolic responses of cyanobacteria‐dominated microbial communities to long‐term soil management practices.

## 4 Discussion

### 4.1 Influence of Long‐Term Agricultural Management on Cyanobacterial Abundance and Structure

This study showed that long‐term agricultural management practices strongly influence soil cyanobacterial abundance, community structure and associated metabolic activity in a semi‐arid Mediterranean olive orchard. The integration of cultivation‐based, molecular and metabolomic approaches provided convergent evidence that sustainable management promotes a more abundant, diverse and functionally oriented cyanobacteria‐enriched bacterial community compared with conventional management. These findings are consistent with the well‐established sensitivity of soil cyanobacteria to physical disturbance, organic matter availability and microclimatic stability (Chung et al. 2019; Hakkoum et al. 2021).

The higher cyanobacterial abundance observed under sustainable management likely reflects the cumulative effects of reduced soil disturbance, continuous organic inputs, permanent soil cover and improved moisture retention. Such conditions favour the establishment and persistence of photoautotrophic microorganisms in surface soil layers, where light availability supports cyanobacterial growth and activity (Chung et al. 2019; Ramakrishnan et al. 2023). In contrast, repeated mechanical tillage, removal of organic residues and rainfed conditions under conventional management likely disrupted cyanobacterial filaments and soil aggregates, limiting the development of structurally complex cyanobacteria‐dominated communities (Crouzet et al. 2019). Similar responses of soil cyanobacteria to tillage intensity and organic matter inputs have been reported across Mediterranean and arid agroecosystems (Isichei 1990; Alghanmi and Jawad 2019).

The predominance of filamentous and heterocyst‐forming cyanobacteria under sustainable management further supports this interpretation. These morphotypes are particularly adapted to stable soil environments, where they can form persistent networks, contribute to soil aggregation through EPS and support nitrogen inputs via biological fixation (Chittora et al. 2020; Bataeva and Grigoryan 2024). Conversely, the simpler community structure associated with conventional management is consistent with strong environmental filtering, favouring taxa capable of rapid colonisation and tolerance of disturbance over long‐term ecological integration.

Multivariate analyses of bacterial communities revealed a clear separation between sustainably and conventionally managed soils, indicating that agricultural management acts as a dominant driver of microbial community assembly. The distinct clustering of samples suggests that long‐term management regimes shape not only cyanobacterial populations but also broader bacterial assemblages enriched under nitrogen‐limited conditions, reinforcing the concept of management‐induced alternative soil microbial states (Yaghoubi Khanghahi et al. 2020; Hsiao et al. 2025).

The association of sustainable management with bacterial phyla commonly linked to nutrient cycling, organic matter turnover and soil structural stability points out to a more functionally interconnected microbial network. The emergence of such highly connected microbial consortia has been widely associated with improved nutrient use efficiency and greater ecosystem multifunctionality and stability, enhancing, for instance, the resilience of soil functions to environmental fluctuations (Torsvik and Øvreås 2002; de Vries et al. 2018; Banerjee et al. 2019; Wagg et al. 2019). In contrast, the bacterial assemblages associated with conventional management showed community patterns consistent with the long‐term use of tillage and mineral fertilisation. Although these factors were not directly quantified in the present study, previous research has shown that recurrent disturbance and mineral‐input‐based management can simplify microbial community organisation and are often associated with reduced network complexity and loss of key interacting taxa (de Vries et al. 2018; Banerjee et al. 2019). Our observations are consistent with studies reporting that reduced tillage and organic inputs promote microbial diversity and functional redundancy, thereby enhancing soil resilience (Yaghoubi Khanghahi et al. 2024; Mishra et al. 2025).

### 4.2 Cyanobacterial Genera as Indicators of Management Intensity

At the genus level, soil management exerted a strong selective pressure on the composition of cyanobacteria within the enriched bacterial assemblages. The dominance of *Leptolyngbya* under conventional management suggests that members of this genus possess adaptive traits that may confer competitive advantages under disturbed conditions. Several *Leptolyngbya* species are filamentous cyanobacteria capable of colonising diverse and environmentally challenging habitats, and have been reported to show broad physiological tolerance, including resistance to desiccation, salinity fluctuations, nutrient limitation and other environmental stresses (Kugler and Dong 2019; Moia et al. 2023; Lee et al. 2024). In contrast, the increased representation of *Nodosilinea* under sustainable management reflects a shift towards taxa better suited to stable environments with continuous organic inputs and lower mechanical disturbance.

The coexistence of heterocystous (*Nostoc*) and non‐heterocystous (*Leptolyngbya*, *Nodosilinea*, *Oscillatoria*) genera across both management systems indicates a degree of functional redundancy in nitrogen acquisition strategies within cyanobacteria‐dominated communities. However, changes in dominance patterns highlight the role of long‐term management in shaping ecological strategies rather than simply determining taxonomic presence. These findings support the use of dominant cyanobacterial genera as sensitive bioindicators of soil management intensity and ecological condition, particularly in semi‐arid agroecosystems (Mateo et al. 2015; Barinova 2025).

### 4.3 Management‐Driven Shifts in Cyanobacterial Metabolic Strategies

Metabolomic analyses revealed that cyanobacteria‐enriched nitrogen‐fixing bacterial communities respond to long‐term soil management through pronounced metabolic reprogramming. The distinct separation between sustainably and conventionally managed soils in multivariate analyses indicates that management practices influence not only community structure but also intracellular and extracellular metabolic pathways within these enriched assemblages.

Under conventional management, cyanobacteria‐associated assemblages showed enhanced biosynthesis of fatty acids, membrane lipids and vitamin‐related compounds. Such lipid remodelling is a well‐documented bacterial stress adaptation mechanism (Chwastek et al. 2020), enabling dominant cyanobacterial taxa to maintain membrane integrity, fluidity and redox balance under conditions of mechanical disturbance and nutrient imbalance. The stimulation of vitamin biosynthesis further suggests increased investment in antioxidant defences and enzymatic protection, consistent with exposure to fluctuating and stressful soil environments (Rani et al. 2021). In contrast, assemblages associated with sustainable management displayed a relative downregulation of stress‐related biosynthetic pathways and a selective enhancement of amino acid metabolism and signalling‐related compounds. This metabolic profile suggests a shift from survival‐oriented strategies towards functional integration within the soil–plant–microbe continuum, reflecting improved environmental stability and resource availability.

One of the most ecologically significant findings is the upregulation of amino acid biosynthesis and hormone‐ and signalling‐related metabolites in cyanobacteria‐dominated assemblages under sustainable management. Amino acids play a central role in nitrogen cycling and can act as readily available substrates for heterotrophic microorganisms and plant roots, thereby strengthening microbial network connectivity and nutrient exchange (González‐López et al. 2005; Moe 2013).

The enhanced activity of phytohormone‐related pathways, including cytokinin biosynthesis and ethylene biosynthesis‐related pathways, is particularly noteworthy. Cyanobacteria are increasingly recognised as contributors to plant growth promotion through the production of bioactive compounds that influence root development, nutrient uptake and stress responses (Jurado‐Flores et al. 2025). The observed hormonal modulation suggests that cyanobacteria‐dominated communities under sustainable management may actively participate in plant–microbe signalling, reinforcing positive feedback between soil microbial communities and olive tree yield.

### 4.4 Extracellular Metabolite Secretion and Ecological Functioning

Differences in extracellular metabolite profiles further underscored the functional divergence between management systems. Under sustainable management, cyanobacteria‐enriched assemblages released distinct sets of metabolites potentially involved in soil aggregation, microbial communication and nutrient mobilisation. Such compounds may contribute to rhizosphere structuring and enhanced soil resilience, consistent with the recognised role of cyanobacteria in biological soil crust formation and stabilisation (Samolov et al. 2020; Novakovskaya et al. 2022). Moreover, cyanobacteria can contribute to the production of a diverse pool of secondary metabolites functioning as bioactive signals‐like, such as polyketides or phenylpropanoids, which can trigger plant defence response activation against pathogens, increasing the ecosystem defensive chemicals and expanding the potential for plant–microbe interactions (Stringlis et al. 2018; Mhlongo et al. 2018).

Conversely, the extracellular metabolomic signature associated with conventional management reflected a metabolic emphasis on stress mitigation rather than ecosystem service provision. The identification of management‐specific extracellular metabolite biomarkers highlighted the potential of cyanobacteria‐associated metabolomic profiles as sensitive indicators of soil health and long‐term management impacts.

### 4.5 Implications for Soil Health and Sustainable Agriculture

Overall, these results indicate that sustainable soil management fosters cyanobacteria‐dominated microbial communities that are not only more abundant but also metabolically oriented towards ecosystem functioning rather than stress tolerance. By contributing to nitrogen cycling, producing bioactive compounds, enhancing soil structure and supporting microbial interactions, these communities may play a pivotal role in improving soil quality and resilience in semi‐arid agricultural systems. In contrast, conventional practices appear to constrain cyanobacteria‐associated functional potential, reinforcing survival‐oriented metabolic pathways at the expense of ecological contribution. These findings support the integration of cyanobacteria‐enriched communities as both bioindicators and functional agents in sustainable agriculture.

### 4.6 Methodological Considerations on (Cyano)bacterial Enrichment and DNA Origin

It is essential to emphasise that the bacterial taxa and metabolic profiles reported in this study are derived from organisms selectively enriched in a modified Bristol liquid medium devoid of combined nitrogen, designed to promote the growth of nitrogen‐fixing cyanobacteria. Consequently, DNA extraction and downstream molecular and metabolomic analyses were performed on biomass grown in this selective medium rather than directly from bulk soil. This approach enabled a targeted investigation of the functional potential of cultivable, cyanobacteria‐dominated nitrogen‐fixing bacterial assemblage**s**, but it does not represent the full in situ soil cyanobacterial community. Therefore, the observed taxonomic and metabolic patterns reflect the responsive fraction of soil bacteria capable of growth under nitrogen‐limited conditions, in which cyanobacteria constitute a dominant component, rather than total soil microbial diversity. Similar enrichment‐based strategies have been widely used to elucidate functional traits of soil cyanobacteria while acknowledging their inherent selectivity (Patil and Singh 2022; Melkonyan et al. 2025).

An additional limitation of this enrichment‐based approach is that cultivation conditions may selectively favour specific cyanobacterial taxa and associated microorganisms that are better adapted to growth in nitrogen‐free Bristol medium. Consequently, fast‐growing and cultivation‐responsive taxa may become overrepresented, whereas slow‐growing, thick‐sheathed, dormant or currently uncultivable cyanobacterial lineages may be underrepresented or absent from the recovered assemblages. Therefore, the community composition described here should be interpreted as the cultivable and enrichment‐responsive fraction of the soil microbiome, not as a comprehensive representation of the in situ cyanobacterial community.

In addition, cyanobacterial abundance was estimated using the MPN method, which provides a cultivation‐dependent and semi‐quantitative measure of the cultivable fraction. MPN estimates are influenced by growth conditions, dilution effects and taxon‐specific cultivation responses (Petri et al. 2019) and therefore should not be interpreted as absolute estimates of total soil cyanobacterial abundance. Future studies could complement this approach with cultivation‐independent methods, such as cyanobacteria‐specific 16S rRNA gene sequencing or quantitative PCR, to better characterise both cultivable and non‐cultivable fractions of the soil cyanobacterial community. Furthermore, the relatively low MPN values observed in this study likely reflect the highly selective nature of the cultivation conditions. Because the assay was based on growth in nitrogen‐free Bristol medium, only the cultivable nitrogen‐fixing fraction capable of responding under the imposed enrichment conditions contributed to the final estimates. Therefore, these values should be interpreted as relative indicators of the abundance of enrichment‐responsive diazotrophic cyanobacteria, and not as absolute estimates of total soil cyanobacterial populations.

## 5 Conclusion

In conclusion, long‐term sustainable agricultural management profoundly shaped soil cyanobacteria‐dominated microbial communities, promoting greater abundance, structural complexity and metabolic orientation towards ecosystem services. The combined use of cultivation‐based, molecular and metabolomic approaches showed that cyanobacteria‐associated assemblages respond to management practices not only taxonomically but also functionally, with important implications for soil health, resilience and sustainable olive production in semi‐arid environments.

## Author Contributions

**Lisa Signorile:** investigation, validation. **Mohammad Yaghoubi Khanghahi:** methodology, software, formal analysis, writing – original draft, writing – review and editing. **Luigi Lucini:** methodology, investigation, validation. **Leilei Zhang:** methodology, software, data curation, investigation, writing – review and editing. **Carmine Crecchio:** conceptualization, writing – review and editing. **Alba N. Mininni:** writing – review and editing, investigation. **Francesco Maria Calabrese:** methodology, software, formal analysis, writing – review and editing. **Rosangela Addesso:** conceptualization, methodology, writing – review and editing, project administration, investigation. **Matteo Bernardi:** methodology, investigation. **Maria De Angelis:** investigation. **Adriano Sofo:** conceptualization, supervision, project administration, resources, writing – review and editing, visualization, funding acquisition. **Mario De Tullio:** investigation, methodology, data curation. **Bartolomeo Dichio:** writing – review and editing, investigation. **Margherita Chiarini:** methodology, investigation.

## Funding

This work was supported by European Union Next‐Generation EU (CN00000022).

## Conflicts of Interest

The authors declare no conflicts of interest.

## Supporting information


**Figure S1:** Mean relative abundances (%) of cyanobacterial taxa detected within the cyanobacteria‐enriched microbial assemblages recovered from conventionally managed (*C*_mng_) and sustainably managed (*S*_mng_) soils.
**Figure S2:** Hierarchical cluster analysis (HCA) of metabolomic profiles of cyanobacteria‐enriched microbial assemblages derived from sustainable (*S*_mng_) and conventional (*C*_mng_) soil management systems.

## Data Availability

The data that support the findings of this study are available on request from the corresponding author. The data are not publicly available due to privacy or ethical restrictions.

## References

[emi70384-bib-0001] Addesso, R. , D. Baldantoni , B. Cubero , et al. 2023. “A Multidisciplinary Approach to the Comparison of Three Contrasting Treatments on Both Lampenflora Community and Underlying Rock Surface.” Biofouling 39: 204–217. 37092276 10.1080/08927014.2023.2202314

[emi70384-bib-0002] Addesso, R. , D. Baldantoni , B. Cubero , et al. 2024. “Unveiling the Menace of Lampenflora to Underground Tourist Environments.” Scientific Reports 14: 20789. 39242666 10.1038/s41598-024-66383-5 PMC11379952

[emi70384-bib-0003] Alghanmi, H. A. , and H. M. Jawad . 2019. “Effect of Environmental Factors on Cyanobacteria Richness in Some Agricultural Soils.” Geomicrobiology Journal 36: 75–84.

[emi70384-bib-0004] Amir, A. , D. McDonald , J. A. Navas‐Molina , et al. 2017. “Deblur Rapidly Resolves Single‐Nucleotide Community Sequence Patterns.” mSystems 2, no. 2: 10–1128. 10.1128/mSystems.00191-16 PMC5340863 28289731

[emi70384-bib-0005] Banerjee, S. , F. Walder , L. Büchi , et al. 2019. “Agricultural Intensification Reduces Microbial Network Complexity and the Abundance of Keystone Taxa in Roots.” ISME Journal 13: 1722–1736. 30850707 10.1038/s41396-019-0383-2 PMC6591126

[emi70384-bib-0006] Barinova, S. 2025. “Database of Ecological Indicators of Freshwater Algae and Cyanobacteria.” Ecology and Diversity 2: 10003.

[emi70384-bib-0007] Bataeva, Y. V. , and L. N. Grigoryan . 2024. “Ecological Features and Adaptive Capabilities of Cyanobacteria in Desert Ecosystems: A Review.” Eurasian Soil Science 57: 430–445.

[emi70384-bib-0008] Bolyen, E. , J. R. Rideout , and M. R. Dillon . 2019. “Reproducible, Interactive, Scalable and Extensible Microbiome Data Science Using QIIME 2.” Nature Biotechnology 37: 852–857. 10.1038/s41587-019-0209-9. PMC7015180 31341288

[emi70384-bib-0009] Cano‐Díaz, C. 2026. “The Unexpected Megadiverse World of Soil Cyanobacteria.” In Aerophytic Algae and Cyanobacteria, 47–68. Academic Press.

[emi70384-bib-0010] Chittora, D. , M. Meena , T. Barupal , P. Swapnil , and K. Sharma . 2020. “Cyanobacteria as a Source of Biofertilizers for Sustainable Agriculture.” Biochemistry and Biophysics Reports 22: 100737. 32083191 10.1016/j.bbrep.2020.100737 PMC7021550

[emi70384-bib-0011] Chung, Y. A. , B. Thornton , E. Dettweiler‐Robinson , and J. A. Rudgers . 2019. “Soil Surface Disturbance Alters Cyanobacterial Biocrusts and Soil Properties in Dry Grassland and Shrubland Ecosystems.” Plant and Soil 441: 147–159.

[emi70384-bib-0012] Chwastek, G. , M. A. Surma , S. Rizk , et al. 2020. “Principles of Membrane Adaptation Revealed Through Environmentally Induced Bacterial Lipidome Remodeling.” Cell Reports 32: 108066. 32966790 10.1016/j.celrep.2020.108165

[emi70384-bib-0013] Crouzet, O. , L. Consentino , J. P. Pétraud , et al. 2019. “Soil Photosynthetic Microbial Communities Mediate Aggregate Stability: Influence of Cropping Systems and Herbicide Use in an Agricultural Soil.” Frontiers in Microbiology 10: 1319. 31258520 10.3389/fmicb.2019.01319 PMC6587365

[emi70384-bib-0014] Gonçalves, A. L. 2021. “The Use of Microalgae and Cyanobacteria in the Improvement of Agricultural Practices: A Review on Their Biofertilising, Biostimulating and Biopesticide Roles.” Applied Sciences 11: 871.

[emi70384-bib-0015] González‐López, J. , B. Rodelas , C. Pozo , V. Salmerón‐López , M. V. Martínez‐Toledo , and V. Salmerón . 2005. “Liberation of Amino Acids by Heterotrophic Nitrogen‐Fixing Bacteria.” Amino Acids 28: 363–367. 15827688 10.1007/s00726-005-0178-9

[emi70384-bib-0016] Hakkoum, Z. , F. Minaoui , M. Douma , K. Mouhri , and M. Loudiki . 2021. “Impact of Human Disturbances on Soil Cyanobacteria Diversity and Distribution in Suburban Arid Area of Marrakesh, Morocco.” Ecological Processes 10: 42.

[emi70384-bib-0017] Hsiao, C. J. , M. Mushtaq , G. F. Sassenrath , L. H. Zeglin , G. M. Hettiarachchi , and C. W. Rice . 2025. “Long‐Term Tillage and Compost Shape Soil Microbes Under Soil Organic Carbon Equilibrium.” European Journal of Soil Science 76: e70125.

[emi70384-bib-0018] Isichei, A. O. 1990. “The Role of Algae and Cyanobacteria in Arid Lands: A Review.” Arid Soil Research and Rehabilitation 4: 1–17.

[emi70384-bib-0053] Jamali, M. , E. Bakhshandeh , M. Yaghoubi Khanghahi , and C. Crecchio . 2021. “Metadata Analysis to Evaluate Environmental Impacts of Wheat Residues Burning on Soil Quality in Developing and Developed Countries.” Sustainability 13, no. 11: 6356. 10.3390/su13116356.

[emi70384-bib-0019] Jurado‐Flores, A. , L. G. Heredia‐Martínez , G. Torres‐Cortes , and E. Díaz‐Santos . 2025. “Harnessing Microalgae and Cyanobacteria for Sustainable Agriculture: Mechanistic Insights and Applications as Biostimulants, Biofertilizers and Biocontrol Agents.” Agriculture 15: 1–3.

[emi70384-bib-0020] Kaehler, B. D. 2022. Silva 138.1 Taxonomy Classifiers for Use With QIIME 2 q2‐Feature‐Classifier. Zenodo.

[emi70384-bib-0021] Kugler, A. , and H. Dong . 2019. “Phyllosilicates as Protective Habitats of Filamentous Cyanobacteria Leptolyngbya Against Ultraviolet Radiation.” PLoS One 14, no. 7: e0219616. 31295311 10.1371/journal.pone.0219616 PMC6623962

[emi70384-bib-0022] Lee, W.‐K. , Y.‐K. Ryu , T. Kim , et al. 2024. “Optimization of Industrial‐Scale Cultivation Conditions to Enhance the Nutritional Composition of Nontoxic Cyanobacterium *Leptolyngbya* sp. KIOST‐1.” Applied Sciences 14, no. 1: 282. 10.3390/app14010282.

[emi70384-bib-0023] Lucius, S. , and M. Hagemann . 2024. “The Primary Carbon Metabolism in Cyanobacteria and Its Regulation.” Frontiers in Plant Science 15: 1417680. 39036361 10.3389/fpls.2024.1417680 PMC11257934

[emi70384-bib-0024] Mateo, P. , F. Leganés , E. Perona , V. Loza , and F. Fernández‐Piñas . 2015. “Cyanobacteria as Bioindicators and Bioreporters of Environmental Analysis in Aquatic Ecosystems.” Biodiversity and Conservation 24: 909–948.

[emi70384-bib-0025] Melkonyan, L. , A. Ferreira , C. R. Bastos , et al. 2025. “Reducing Nutrient Requirement Using Nitrogen‐Fixing Bacteria for Microalgae Cultivation.” Bioresource Technology Reports 31: 102180.

[emi70384-bib-0026] Metting, B. 1981. “The Systematics and Ecology of Soil Algae.” Botanical Review 47: 195–312.

[emi70384-bib-0027] Mhlongo, M. I. , L. A. Piater , P. A. Steenkamp , N. E. Madala , and I. A. Dubery . 2018. “Metabolomic Profiling of Plant Defense Responses to Pathogen Infection.” Frontiers in Plant Science 9: 1123.30131818

[emi70384-bib-0028] Mishra, A. K. , P. Yadav , S. Sharma , and P. Maurya . 2025. “Comparison of Microbial Diversity and Community Structure in Soils Managed With Organic and Chemical Fertilization Strategies Using Amplicon Sequencing of 16S and ITS Regions.” Frontiers in Microbiology 15: 1444903. 40017465 10.3389/fmicb.2024.1444903 PMC11865238

[emi70384-bib-0029] Moe, L. A. 2013. “Amino Acids in the Rhizosphere: From Plants to Microbes.” American Journal of Botany 100: 1692–1705. 23956051 10.3732/ajb.1300033

[emi70384-bib-0030] Moia, I. C. , S. B. Pereira , P. Domizio , R. De Philippis , and A. Adessi . 2023. “ *Phormidium ambiguum* and *Leptolyngbya ohadii* Exopolysaccharides Under Low Water Availability.” Polymers 15, no. 8: 1889. 10.3390/polym15081889. 37112036 PMC10142279

[emi70384-bib-0031] Nandagopal, P. , A. N. Steven , L. W. Chan , Z. Rahmat , H. Jamaluddin , and N. I. Mohd Noh . 2021. “Bioactive Metabolites Produced by Cyanobacteria for Growth Adaptation and Their Pharmacological Properties.” Biology 10: 1061. 34681158 10.3390/biology10101061 PMC8533319

[emi70384-bib-0032] Nawaz, T. , S. Fahad , L. Gu , L. Xu , and R. Zhou . 2025. “Harnessing Nitrogen‐Fixing Cyanobacteria for Sustainable Agriculture: Opportunities, Challenges, and Implications for Food Security.” Nitrogen 6: 16.

[emi70384-bib-0033] Novakovskaya, I. V. , E. N. Patova , Y. A. Dubrovskiy , A. B. Novakovskiy , and E. E. Kulyugina . 2022. “Distribution of Algae and Cyanobacteria of Biological Soil Crusts Along the Elevation Gradient in Mountain Plant Communities at the Northern Urals (Russian European Northeast).” Journal of Mountain Science 19: 637–646.

[emi70384-bib-0034] Nowruzi, B. , and M. Alibabaei . 2026. “The Biogeography of Terrestrial Algae: Global Patterns and Local Variations.” In Aerophytic Algae and Cyanobacteria, 31–45. Academic Press.

[emi70384-bib-0036] Pandey, S. , A. Dadsena , P. Shrivastava , et al. 2025. “Cyanobacteria's Advanced Nutrient Homeostasis Strategy Under Limiting Conditions.” In Cyanobacterial Response to Extreme Environments: A Molecular Understanding for a Sustainable Future, 76–100. CABI.

[emi70384-bib-0037] Patil, K. , and D. M. Singh . 2022. “Optimization of Culture Media for the Growth of Anabaena PCC 550, Anabaena PCC 574, Cylindrospermum PCC 518 and Cylindrospermum PCC 567.” Journal of Advanced Scientific Research 13: 106–110.

[emi70384-bib-0038] Penna, D. D. , G. Q. Romero , M. P. Nessel , A. L. Gonzalez , and V. M. Oliveira . 2025. “Bacterial Communities as Bioindicators of Climate Change in Freshwater Ecosystems: Tank Bromeliads as Model Systems.” Ecological Indicators 171: 113161.

[emi70384-bib-0039] Petri, B. , S. R. Chaganti , P. S. Chan , and D. Heath . 2019. “Phytoplankton Growth Characterization in Short‐Term MPN Culture Assays Using 18S Metabarcoding and qRT‐PCR.” Water Research 164: 114941. 31398632 10.1016/j.watres.2019.114941

[emi70384-bib-0040] Ramakrishnan, B. , N. R. Maddela , K. Venkateswarlu , and M. Megharaj . 2023. “Potential of Microalgae and Cyanobacteria to Improve Soil Health and Agricultural Productivity: A Critical View.” Environmental Science: Advances 2: 586–611.

[emi70384-bib-0041] Rani, A. , K. C. Saini , F. Bast , et al. 2021. “Microorganisms: A Potential Source of Bioactive Molecules for Antioxidant Applications.” Molecules 26: 1142. 33672774 10.3390/molecules26041142 PMC7924645

[emi70384-bib-0042] Saleem, A. , S. Anwar , S. Saud , T. Kamal , S. Fahad , and T. Nawaz . 2025. “Cyanobacteria Diversity and Ecological Roles: Insights Into Cyanobacterial Adaptations and Environmental Implications.” Journal of Umm Al‐Qura University for Applied Sciences: 1–9. 10.1007/s43994-025-00261-2.

[emi70384-bib-0043] Samolov, E. , K. Baumann , B. Büdel , et al. 2020. “Biodiversity of Algae and Cyanobacteria in Biological Soil Crusts Collected Along a Climatic Gradient in Chile Using an Integrative Approach.” Microorganisms 8: 1047. 32674483 10.3390/microorganisms8071047 PMC7409284

[emi70384-bib-0044] Sharma, S. , S. Chakraborty , V. Sindhu , A. K. Mishra , and S. S. Singh . 2025. “Elucidating the Role of Compatible Solutes in Growth Protection and Amelioration of Oxidative Stress in the Thermophilic Cyanobacterium Mastigocladus sp. TA‐8 During Temperature Shifts.” Extremophiles 29: 40. 41182410 10.1007/s00792-025-01406-1

[emi70384-bib-0045] Solomon, W. , L. Mutum , M. Rakszegi , T. Janda , and Z. Molnár . 2023. “Harnessing the Synergy of the Cyanobacteria‐Plant Growth‐Promoting Bacteria for Improved Maize ( *Zea mays* ) Growth and Soil Health.” Sustainability 15, no. 24: 16660.

[emi70384-bib-0046] Stringlis, I. A. , K. Yu , K. Feussner , et al. 2018. “MYB72‐Dependent Coumarin Exudation Shapes Root Microbiome Assembly to Promote Plant Health.” Annual Review of Phytopathology 56: 559–586. 10.1073/pnas.1722335115 PMC5984513 29686086

[emi70384-bib-0047] Tan, C. Y. , I. C. Dodd , J. E. Chen , et al. 2021. “Regulation of Algal and Cyanobacterial Auxin Production, Physiology, and Application in Agriculture: An Overview.” Journal of Applied Phycology 33: 2995–3023.

[emi70384-bib-0048] Teplitski, M. , and S. Rajamani . 2010. “Signal and Nutrient Exchange in the Interactions Between Soil Algae and Bacteria.” In Biocommunication in Soil Microorganisms, 413–426. Springer.

[emi70384-bib-0049] Torsvik, V. , and L. Øvreås . 2002. “Microbial Diversity and Function in Soil: From Genes to Ecosystems.” Current Opinion in Microbiology 5: 240–245. 12057676 10.1016/s1369-5274(02)00324-7

[emi70384-bib-0050] de Vries, F. T. , R. I. Griffiths , M. Bailey , et al. 2018. “Soil Bacterial Networks Are Less Stable Under Drought Than Fungal Networks.” Nature Communications 9: 3033. 10.1038/s41467-018-05516-7 PMC6072794 30072764

[emi70384-bib-0051] Wagg, C. , K. Schlaeppi , S. Banerjee , E. E. Kuramae , and M. G. A. van der Heijden . 2019. “Fungal‐Bacterial Diversity and Microbiome Complexity Predict Ecosystem Functioning.” Nature Communications 10: 4841. 10.1038/s41467-019-12798-y PMC6813331 31649246

[emi70384-bib-0054] Yaghoubi Khanghahi, M. , G. Cucci , G. Lacolla , L. Lanzellotti , and C. Crecchio . 2020. “Soil Fertility and Bacterial Community Composition in a Semiarid Mediterranean Agricultural Soil Under Long‐Term Tillage Management.” Soil Use and Management 36: 604–615. 10.1111/sum.12645.

[emi70384-bib-0055] Yaghoubi Khanghahi, M. , M. Curci , E. Cazzato , et al. 2024. “Shifts in Soil Bacterial Communities Under Three‐Year Fertilization Management and Multiple Cropping Systems.” Soil Systems 8, no. 1: 5. 10.3390/soilsystems8010005.

[emi70384-bib-0052] Zhou, X. , B. Liang , T. Zhang , Q. Xiong , X. Ma , and L. Chen . 2024. “Co‐Inoculation of Fungi and Desert Cyanobacteria Facilitates Biological Soil Crust Formation and Soil Fertility.” Frontiers in Microbiology 15: 1377732. 38650889 10.3389/fmicb.2024.1377732 PMC11033444

