# Supplementary material for: Abundance, Diversity and Metabolomic Profiles of Soil Cyanobacteria‐Dominated Microbial Communities Under Long‐Term Differential Management in an Olive Orchard
Source: Environ Microbiol. 2026 Jul 15;28(7):e70384. doi: 10.1111/1462-2920.70384 (PMC13370714; doi:10.1111/1462-2920.70384)
Supplement: Supplementary file 1 — Figure S1: Mean relative abundances (%) of cyanobacterial taxa detected within the cyanobacteria‐enriched microbial assemblages recovered from conventionally managed (C mng) and sustainably managed (S mng) soils. Figure S2: Hierarchical cluster analysis (HCA) of metabolomic profiles of cyanobacteria‐enriched microbial assemblages derived from sustainable (S mng) and conventional (C mng) soil management systems. [file EMI-28-e70384-s001.docx]

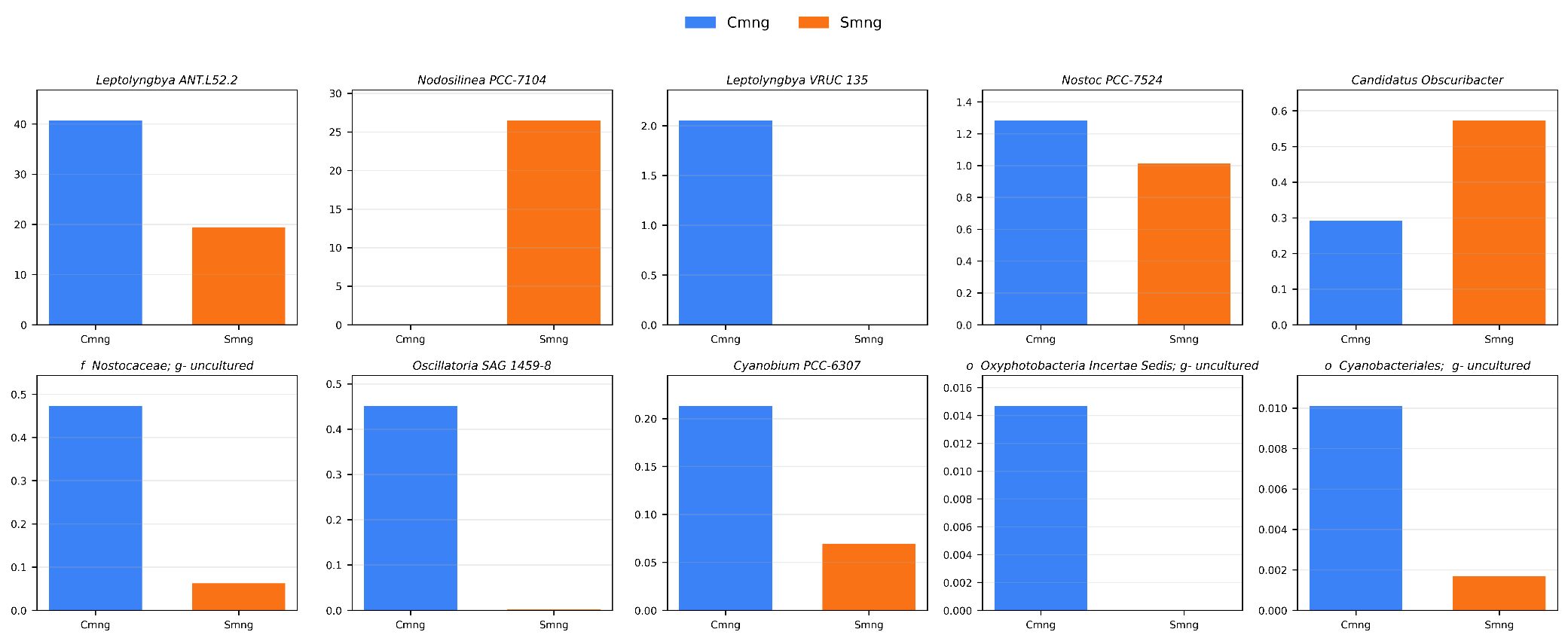


**SUPPLEMENTARY FIGURE S1.** Mean relative abundances (%) of cyanobacterial taxa detected within the cyanobacteria-enriched microbial assemblages recovered from conventionally managed (C_mng_) and sustainably managed (S_mng_) soils. Values represent the average relative abundance across three biological replicates for each management system.

**
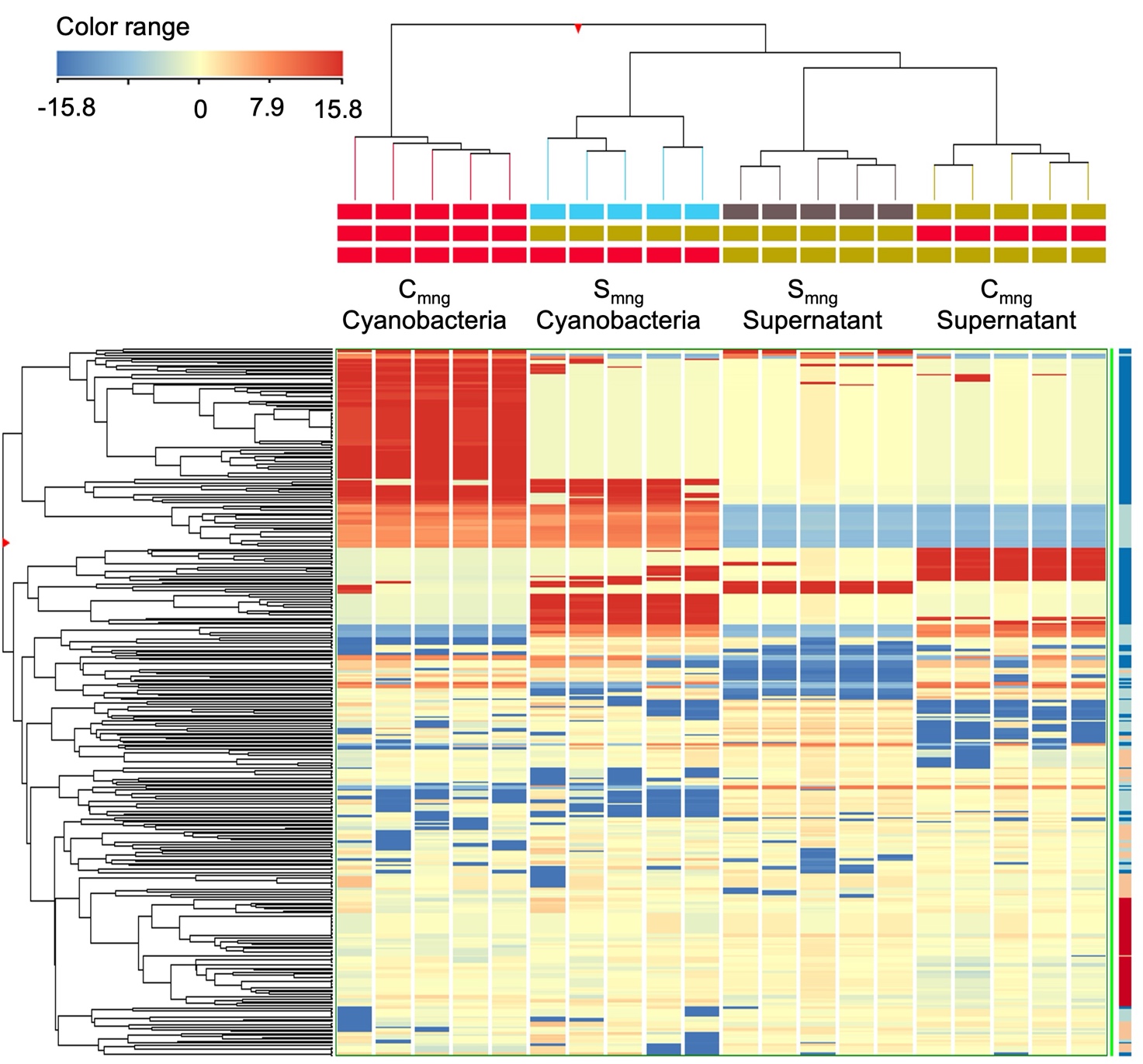
**

**SUPPLEMENTARY FIGURE S2.** Hierarchical cluster analysis (HCA) of metabolomic profiles of cyanobacteria-enriched microbial assemblages derived from sustainable (S_mng_) and conventional (C_mng_) soil management systems.
